# LINE1-mediated epigenetic repression of androgen receptor transcription causes androgen insensitivity syndrome

**DOI:** 10.1038/s41598-024-65439-w

**Published:** 2024-07-15

**Authors:** Jelena Pozojevic, Radhika Sivaprasad, Joshua Laß, Franziska Haarich, Joanne Trinh, Naseebullah Kakar, Kristin Schulz, Kristian Händler, Annemarie A. Verrijn Stuart, Jacques C. Giltay, Koen L. van Gassen, Almuth Caliebe, Paul-Martin Holterhus, Malte Spielmann, Nadine C. Hornig

**Affiliations:** 1 Institute of Human Genetics, University of Lübeck and University Hospital Schleswig-Holstein, Lübeck, Germany; 2 Institute of Human Genetics, University of Kiel and University Hospital Schleswig-Holstein, Kiel, Germany; 3 Institute of Neurogenetics, University of Lübeck and University Hospital Schleswig-Holstein, Lübeck, Germany; 4 Institute of Cardiogenetics, University of Lübeck and German Centre for Cardiovascular Research (DZHK), Partner Site Hamburg/Lübeck/Kiel, Lübeck, Germany; 5 Department of Pediatric Endocrinology, Wilhelmina Children’s Hospital, University Medical Center Utrecht, Utrecht, The Netherlands; 6 Division Laboratories, Pharmacy and Biomedical Genetics, University Medical Center Utrecht, Utrecht, The Netherlands; 7 Department of Genetics, University Medical Center Utrecht, Utrecht, The Netherlands; 8 Division of Paediatric Endocrinology and Diabetes, Department of Paediatrics, University Hospital Schleswig-Holstein, Kiel, Germany; 9 Human Molecular Genomics Group, Max Planck Institute for Molecular Genetics, Berlin, Germany; 10 German Centre for Cardiovascular Research (DZHK), Partner Site Hamburg/Lübeck/Kiel, Lübeck, Germany; 11 Department of Biotechnology, FLS&I, BUITEMS, Quetta, Pakistan

**Keywords:** DNA, Hormones, Diseases

## Abstract

Androgen insensitivity syndrome (AIS) is a difference of sex development (DSD) characterized by different degrees of undervirilization in individuals with a 46,XY karyotype despite normal to high gonadal testosterone production. Classically, AIS is explained by hemizygous mutations in the X-chromosomal androgen receptor (*AR*) gene. Nevertheless, the majority of individuals with clinically diagnosed AIS do not carry an *AR* gene mutation. Here, we present a patient with a 46,XY karyotype, born with undervirilized genitalia, age-appropriate testosterone levels and no uterus, characteristic for AIS. Diagnostic whole exome sequencing (WES) showed a maternally inherited LINE1 (L1) retrotransposon insertion in the 5′ untranslated region (5′UTR) of the *AR* gene. Long-read nanopore sequencing confirmed this as an insertion of a truncated L1 element of ≈ 2.7 kb and showed an increased DNA methylation at the L1 insertion site in patient-derived genital skin fibroblasts (GSFs) compared to healthy controls. The insertion coincided with reduced AR transcript and protein levels in patient-derived GSFs confirming the clinical diagnosis AIS. Our results underline the relevance of retrotransposons in human disease, and expand the growing list of human diseases associated with them.

## Introduction

Androgen insensitivity syndrome (AIS) is a common cause of 46,XY differences of sex development (DSD) characterized by reduced or absent cellular response to androgens. The androgens testosterone and dihydrotestosterone (DHT) act through the androgen receptor (AR), and androgen action is necessary for male sex development. Mutations in the X-chromosomal *AR* gene therefore lead to an incomplete virilization or a female genital phenotype at birth despite a 46,XY karyotype and androgen producing testes, characteristic for AIS. AIS can be divided into a mild, partial and complete form. The mild form (MAIS) is associated with gynecomastia and/or infertility, the partial form (PAIS) covers a broader phenotypic spectrum from micropenis to severe hypospadias, while the complete form (CAIS) is associated with female outer genitalia, no uterus and undescended androgen-producing testes. Over 100 mutations within the coding region of the *AR* have been shown to cause AIS with few descriptions of non-coding variants^1^. These comprise deep intronic mutations and mutations in the 5′ untranslated region (5′UTR) of the *AR*^2,3^. 5′UTRs are the sites where ribosomes enter and scan the mRNA for a suitable translational start codon at which the translation initiation complex becomes assembled. Depending on their length, they can contain cis-regulatory elements such as upstream open reading frames (uORFs) and secondary structures that regulate mRNA stability and gene expression both on the transcriptional and the translational level^4,5^. The 5′UTR of the *AR* is 1115 nucleotides (nt) long, exceeding the typical length of 100–220 nt across species and GC-rich, thus prone to secondary structures indicative for gene expression control. In line with this, we previously described two patients with CAIS and a recurrent mutation in the 5′UTR of the *AR* creating a translated uORF interfering strongly with AR protein expression^3^. The *AR* 5′UTR also contains two short simple sequence repeats but is devoid of transposable elements, e.g., retrotransposons. Long interspersed nuclear elements 1 (LINE1 or L1) belong to the family of retrotransposons that perpetuate through a “copy and paste” mechanism in the genome and comprise approximately 17% of the human DNA^6^. Human L1 encodes two proteins, ORF1p and ORF2p, the latter having endonuclease (EN) and reverse transcriptase (RT) activities^7,8^ necessary for retrotransposition. The majority of L1 copies are truncated, fragmented, or mutated and lost their ability to retrotranspose^9^. Repeat insertions in promoters are important modulators of gene expression and are known to enhance or repress transcription by steroid receptors^10–12^. In prostate cancer cells, LINE-1 ORF-1p has been shown to act as a co-activator of the androgen receptor promoting cell proliferation^13^.

We here report a patient with PAIS and an insertion of a L1 element in the 5′UTR of the *AR*. We show that this insertion causes a strongly reduced *AR* mRNA expression both through the disruption of regulatory sequences in the 5′UTR and through DNA methylation at the insertion site, thus explaining the clinical phenotype.

## Results

### Clinical data

The here described child was born at term to non-consanguineous parents (birth weight 4470 g). A structural ultrasound at 20 weeks of gestation raised some uncertainty about the external genitalia, thereafter assessment varied. However, no prenatal tests were done. Pregnancy was otherwise uneventful. At birth, sex assignment was deferred due to perineal hypospadias. Labioscrotal folds were visible, without gonadal tissue palpable in these folds. A genital tubercle (< 1 cm) was visible between unfused labioscrotal folds with a perineal urethral meatus. The anogenital distance was not reported. Prader stage was II–III; external masculinization score (EMS) score 0–1^14^. Karyotyping revealed a 46,XY karyotype. Endocrine analysis (blood and urine) and a human chorionic gonadotropin (hCG) test at 4 weeks of age did not show signs of disturbed testosterone synthesis and did indicate the presence of male gonadal tissue (Table 1). The T/DHT ratio was normal, indicating normal conversion of T into DHT by 5-alpha reductase activity^15^. Genitography and ultrasound 1 day after birth as well as laparoscopy at age 1.5 years did not show signs of Mullerian structures or a rudimentary vagina. Androgen insensitivity was deemed the most probable diagnosis. As there was very little virilization without palpable gonads, female gender assignment was decided upon after a period of intensive parental counseling. Single gene genetic analyses did not reveal abnormalities in *AR*, *SRD5A2*, *HSD17B3*, *WT1*, *NR5A1*, *MAP3K1* and no *DAX* duplication was detected. Laparoscopy at age 1.5 years did show a bilateral inguinal hernia containing gonadal tissue with testicular appearance. Bilateral gonadal biopsies showed testicular tissue without abnormalities and no signs of (pre-) malignancy. Immunohistology showed no OCT 3/4 positive cells, no CD117 or PLAP positive cells and a positive inhibin B staining of the Sertoli cells in the tubuli seminiferi. A biopsy of labioscrotal skin was taken for further analyses. Currently, aged 10 the patient shows normal linear growth within her target height range when assessed on a female growth chart.

**Table 1:** Steroid hormone concentrations.

Age	Day of birth	1 month (hCG test day1)	1 month (hCG test day4)	9y 2 months (Tanner: A1, P1)	10y 8 months (Tanner: A1, P1)
AMH (pmol/L)	1190			128.52	549.78
17OHP (nmol/L)	65	9.0	13		
Androstenedione (nmol/L)	3.3	1.4	2.8		0.75
Testosterone (nmol/L)	8.4	9.6	24	< 0.20	0.24
DHT (nmol/L)		2.0	4.1		
Progesterone (nmol/L)		4.3			
SHBG (nmol/L)					82
Inhibin B (ng/L)		247			
LH (IU/L)		2.5	1.0	< 0.20	0.60
FSH (IU/L)		1.6	0.62	4.2	5.0
Estradiol (pmol/L)				< 40	< 40
DHEA (nmol/L)				6	3
DHEAS (µmol/L)				3.8	6.4

All results (apart from the HCG test) are obtained in the absence of sex hormone treatment and interpreted in comparison with male normal reference values^16,17^.

HCG test: 1500 IE per day on 3 subsequent days; Interpretation of results on day three of HCG test: normal androstenedione/testosterone ratio; normal testosterone/DHT ratio; sufficient increase of testosterone after HCG. *AMH* Anti-Mullerian hormone, *17OHP* 17-OH-Progesteron, *DHT* dihydrotestosterone, *LH* Luteinizing hormone, *FSH* Follicle stimulating hormone, *DHEA* Dehydroepiandrosterone, *DHEAS* Dehydroepiandrosterone-sulfate.

### AR activity and expression in patient-derived genital skin fibroblasts (GSFs)

**Figure 1:**
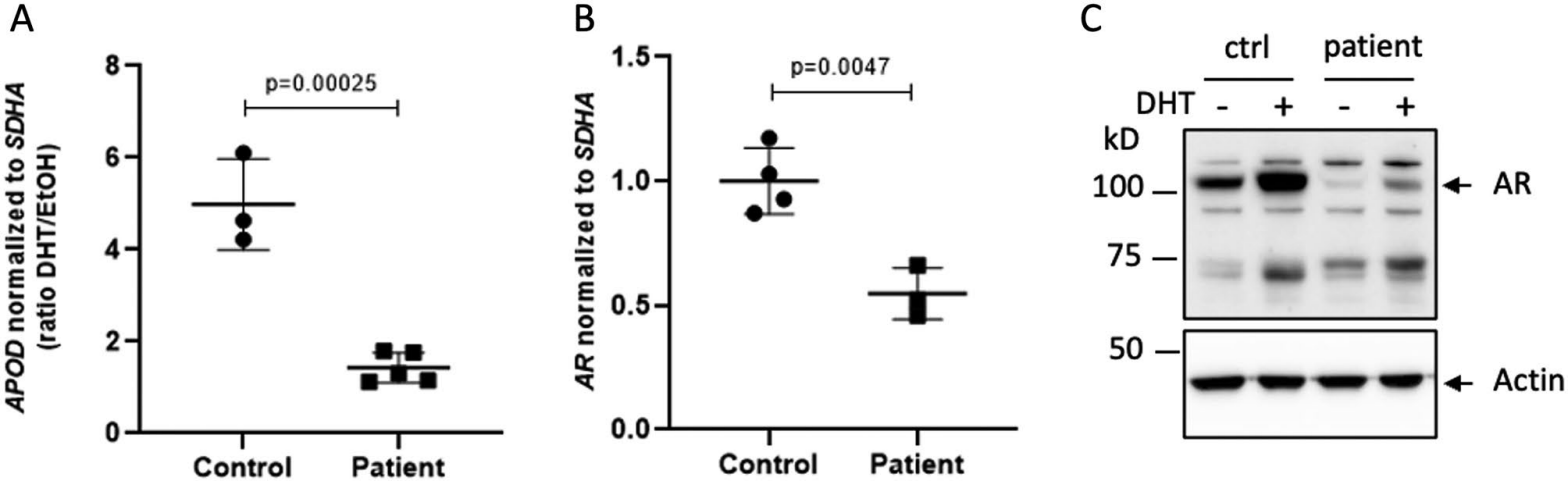
*AR* expression and activity in patient-derived genital skin fibroblasts (GSFs). (**A**) Levels of Apolipoprotein D (*APOD*) mRNA normalized to Succinate Dehydrogenase Complex Flavoprotein Subunit A (*SDHA*). (**B**) *AR* mRNA levels normalized to *SDHA*. (**C**) AR protein levels in control GSFs 1 (ctrl) and patient GSFs, without (−) or with (+) treating the cells with dihydrotestosterone (DHT). Actin served as a loading control.

As the clinical findings strongly suggested androgen insensitivity, we decided to analyze AR function in its target tissue. For this purpose, we treated patient-derived GSFs and male control GSFs with DHT and analyzed the androgen-induced transcription of the AR target gene Apolipoprotein D (*APOD*). This revealed a strong reduction of AR transcriptional activity in patient-derived GSFs (Fig. 1A) as compared to control GSFs. *AR* mRNA expression analysis showed a significant reduction in *AR* mRNA levels (Fig. 1B). This reduction was even stronger on the protein level, and there was very little AR protein expression even after DHT treatment, indicating that the reduced AR activity was due to a reduced *AR* expression (Fig. 1C).

### Genetic analyses

**Figure 2:**
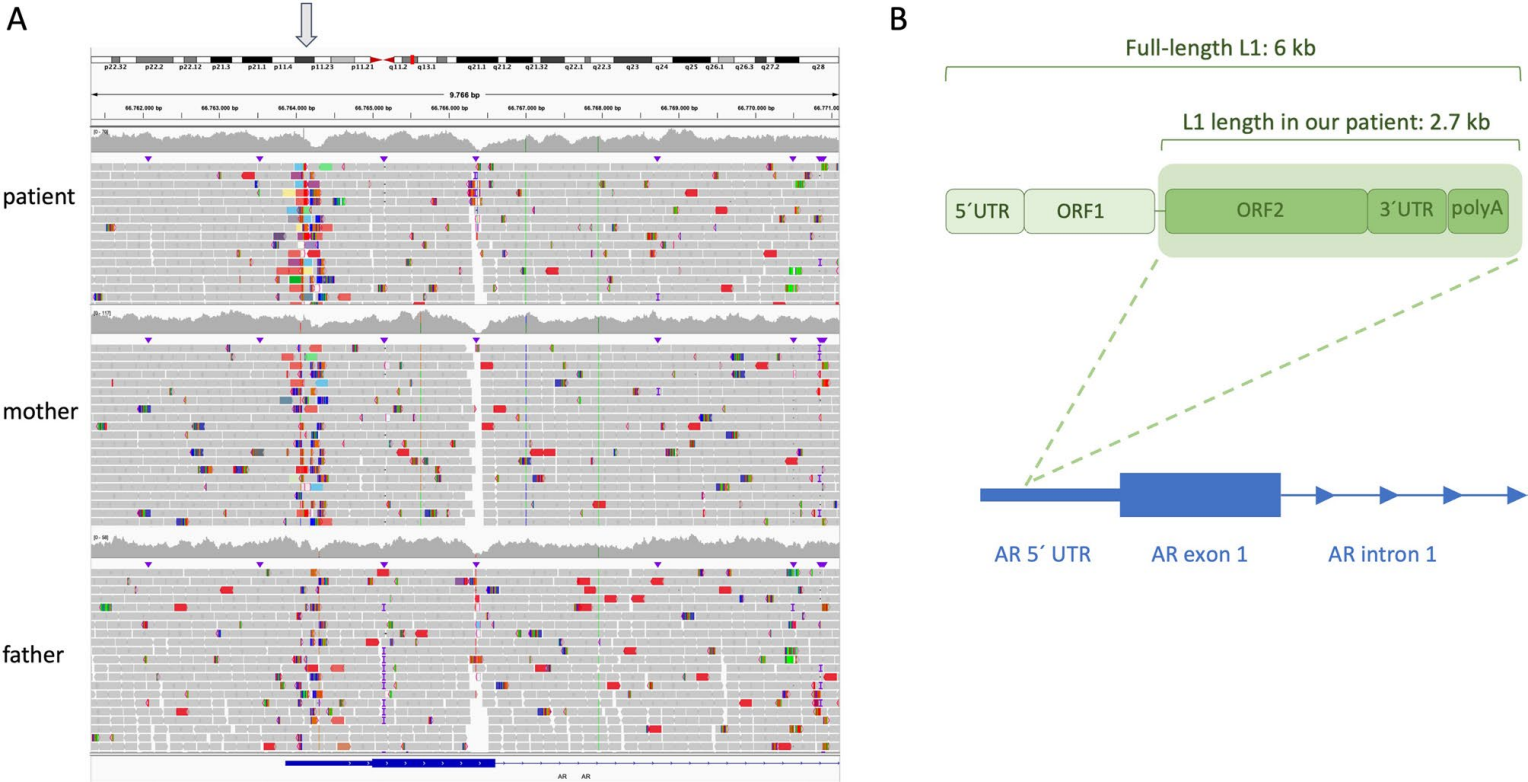
Whole genome sequencing (WGS) shows a maternally inherited L1 insertion in the 5′UTR of the *AR*. (**A**) Integrative Genomics Viewer (IGV) screenshot showing the hemizygous insertion in the patient, inherited from the heterozygous mother, in the *AR* 5′UTR. The insertion site is indicated by an arrow. (**B**) Truncated L1 element in our patient as compared to a full-length L1 element. Individual L1 domains are also depicted in the figure, along with the insertion location in the *AR* 5′UTR.

As sequencing of the *AR* coding region did not reveal any changes, diagnostic whole exome sequencing on blood-derived DNA from the patient was performed. We detected a long interspersed nuclear element (LINE1 or L1) retrotransposon insertion in the *AR* 5′ UTR region (chrX:67,544,266; GRCh38), while its exons and splice-site regions were unaffected. No other pathogenic or likely pathogenic variants were found in the coding regions of other DSD-relevant genes. PCR confirmation and segregation showed that the L1 variant was maternally inherited and that it had occurred de novo in the mother (Supplementary Fig. 1). This result was also confirmed by whole genome sequencing and a long-range PCR, which further revealed the estimated length of the L1 element to be ~ 2.8 kb (Fig. 2A; Supplementary Fig. 2A). To further resolve the complete L1 sequence in the patient, we used long-read nanopore sequencing and found a truncated L1 element consisting of 2752 bp (Supplementary File 1) in contrast to ~ 6 kb full-length L1 elements (Fig. 2B). Sequence similarity alignment revealed that this L1 element is 5′ truncated, consisting of open reading frame 2 (ORF2) that encodes for endonuclease and reverse transcriptase, as well as of a 3′ UTR and a polyA tail. Furthermore, we found that the genomic location of the insertion corresponds to the 5′-TTTT/A-3′ sequence, in line with the fact that ORF2 endonuclease nicks genomic DNA at this specific sequence^8,18,19^. Of note, other maternal family members did not carry the insertion (Supplementary Fig. 1).

### Plasmid-based assays for in vitro expression analyses

**Figure 3:**
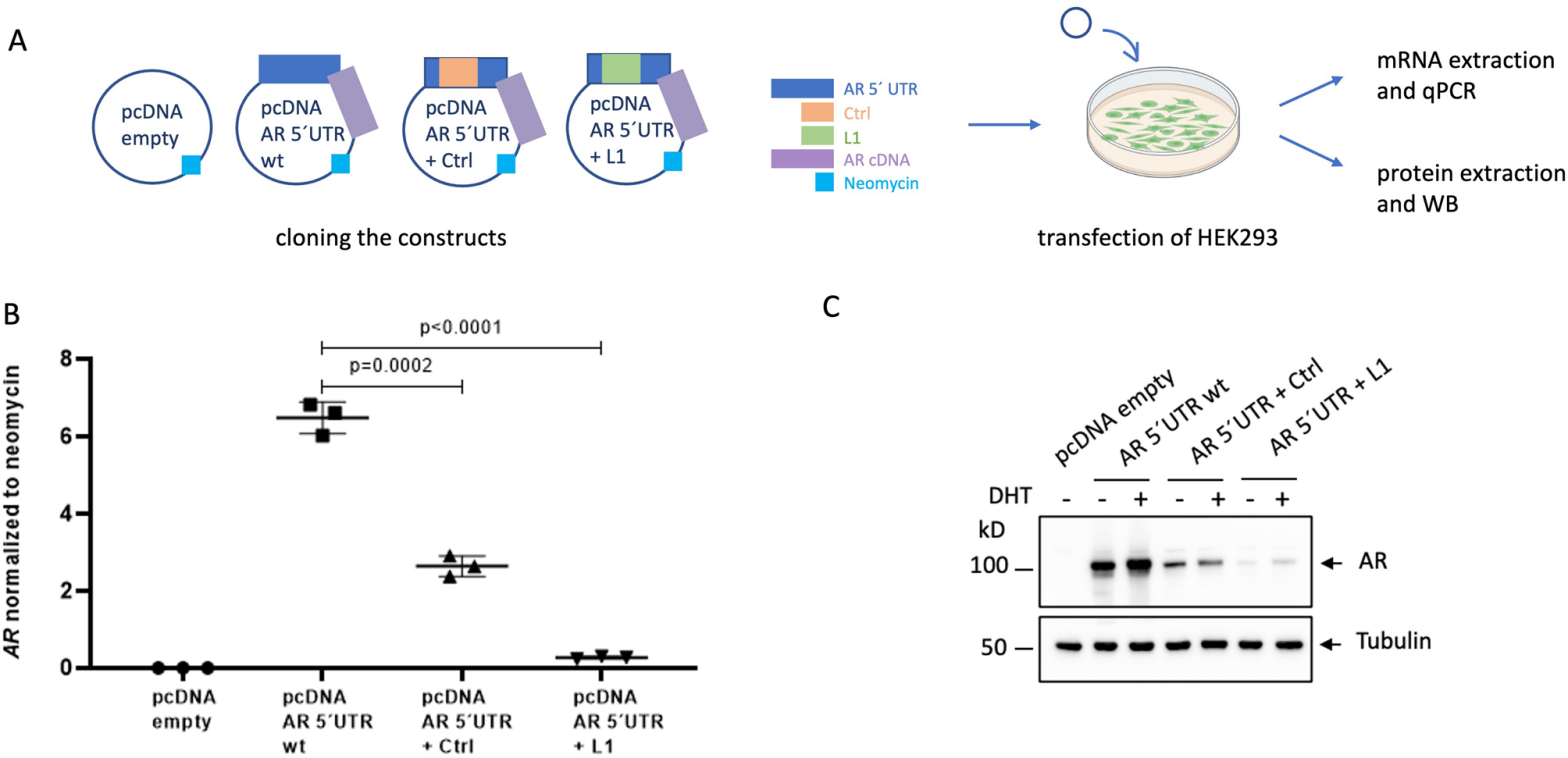
Plasmid-based assays show effects of the L1 insertion on *AR* expression in HEK293 cells. (**A**) constructs created for individual transfections, and experimental workflow. (**B**) *AR* mRNA levels normalized to neomycin (which is encoded by the plasmid). (**C**) AR protein levels in HEK293 transfected with the individual constructs, either with (+) or without (−) treatment with dihydrotestosterone (DHT). Tubulin served as a loading control. WB: Western Blot.

To further investigate whether this L1 retrotransposon insertion is indeed the disease-causing event in our patient, we used plasmid-based assays to assess its effects on *AR* expression. Therefore, either the patient-derived L1 element or a size-matched control (Ctrl) was inserted into a pcDNA vector that contains the *AR* 5′ untranslated region (*AR* 5′UTR) and *AR* coding DNA sequence (*AR* cDNA). Specifically, each of these sequences (L1 or Ctrl) was inserted in the *AR* 5′UTR, at the same location where the L1 element is inserted in our patient, and HEK293 cells were transfected with these constructs for further expression analyses (Fig. 3A). *AR* mRNA levels were found to be decreased in the cells transfected with the constructs that interrupt the wild type (wt) *AR* 5′UTR sequence, with the L1 insertion causing a more severe effect than the size-matched control (Fig. 3B). These results were recapitulated on the protein level, where the L1 insertion decreased AR protein levels again more severely than the size-matched control (Fig. 3C), indicating an additional mechanism to solely promoter sequence disruption.

### Assessment of DNA methylation

**Figure 4:**
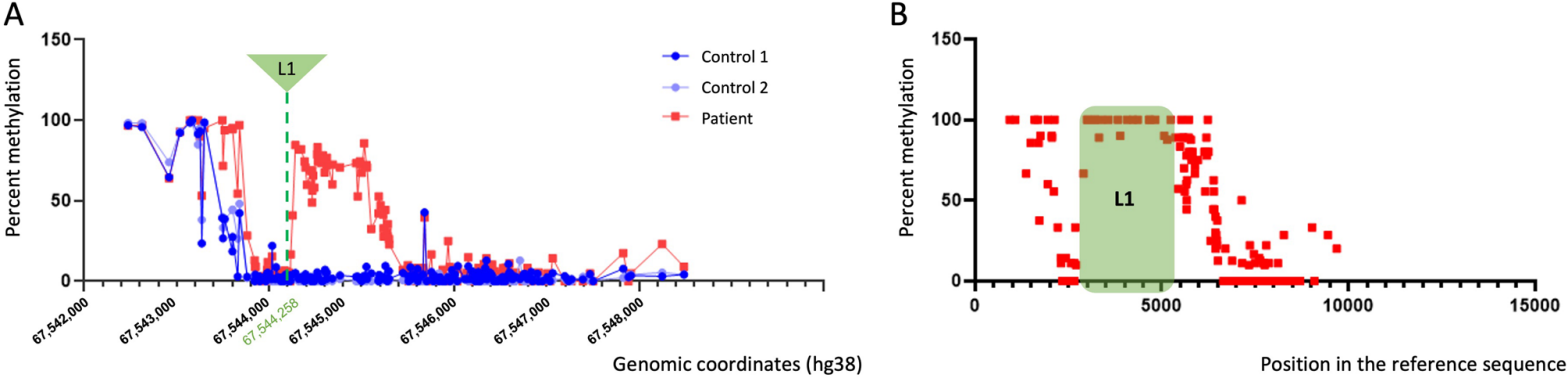
DNA methylation in patient-derived GSFs. (**A**) percent methylation at each individual CpG site in the *AR* 5′UTR of the patient (excluding the L1 insertion) and two healthy controls. Genomic coordinates on the xaxis are given in the hg38 assembly and include only the wild type sequence in the patient and two healthy controls. (**B**) Percent methylation at individual CpG sites in patient-derived cells, including the L1 insertion (in green) and the adjacent regions. Position of the CpGs in the region of interest sequenced by nanopore is given on the xaxis.

Since we observed reduced *AR* expression both in the patient-derived GSFs and in the plasmid-based assays due to the insertion in the *AR* 5′UTR, and our plasmid-based experiments showed that the L1 insertion decreased AR levels stronger than the size-matched control, we aimed to further decipher these mechanisms and hypothesized that these changes might coincide with changes in *AR* 5′UTR DNA methylation caused by the L1 insertion. Transposable elements can change the epigenetic landscape surrounding their insertion, and high DNA methylation of a promoter region is generally associated with reduced levels of gene expression^20,21^. Assessment of native DNA modifications regarding CpG methylation was done with nanopore Cas9-targeted sequencing, where the region of interest included *AR* 5′UTR upstream and downstream sequences of the location of the L1 insertion, in patient-derived and two healthy control GSFs (Supplementary Fig. 2B). DNA methylation was found to be increased in patient-derived GSFs as compared to healthy controls, both upstream and downstream adjacent to the L1 insertion (Fig. 4A). Furthermore, the L1 element itself was highly methylated in patient-derived GSFs, suggesting that host-defense mechanisms are effectively silencing its activity (Fig. 4B).

## Discussion

Over 50% of individuals with the clinical diagnosis AIS do not harbor mutations within the coding region of the *AR* gene. This significantly complicates the counseling and clinical care of these individuals and raises fundamental questions such as sex assignment^1^. GSFs are a valuable tool to evaluate variants outside the *AR* coding region as they are derived from androgen-responsive tissue and thus can be used to assess AR function^22^. Importantly, they also allow for epigenetic analysis of the *AR* genomic region. We previously identified a proximal *AR* promoter region that when epigenetically silenced through DNA-methylation significantly decreased *AR* mRNA expression causing AR-Mutation-Negative Androgen Insensitivity Syndrome (AIS Type II)^23^. Here we report a patient with an L1 insertion in the 5′UTR of the *AR* causative for AIS. This is the second report of a patient with AIS caused by a L1 insertion in the *AR* 5′UTR. Batista et al. reported a large family where an L1 insertion co-segregated with AIS^24^. Affected family members had severe undervirilization of the external genitalia at birth and showed no signs of virilization during puberty. The identified 5′ truncated L1 was 804 nt long, thus smaller than the insertion in our patient and occurred at position c.-268 as compared to position c.-881 in our patient. Forearm-derived skin fibroblasts showed reduced *AR* mRNA expression in one affected family member of the Batista et al. study, indicating a relationship between L1 insertion and *AR* expression. We here elaborated the epigenetic consequences of the L1 insertion and analyzed the mechanism leading to the reduced *AR* expression. We show that an insertion in the *AR* 5′UTR per se can reduce *AR* mRNA expression or stability in vitro, likely due to the disruption of regulatory sequences. Specific insertion of the truncated L1 element reduced *AR*/AR (mRNA and protein, respectively) expression even further, indicating that L1 insertion triggered transcriptional silencing rather than further mRNA instability probably due to DNA methylation, as we observed high DNA methylation in GSF-derived patient DNA surrounding the insertion site.

The L1 insertion in our patient is in line with the well-defined knowledge on L1 elements: (i) the insertion occurred at a 5′-TTTT/A-3′ genomic DNA sequence; (ii) it lacks the 5′ end (ORF1) and indeed the majority of L1 insertions are 5′-truncated; (iii) it is inserted at a low nucleosome occupancy site (i.e., promoter region)^19,25–28^. L1 insertions into coding or regulatory regions have the potential to profoundly alter gene expression and have been associated with various diseases^9^. In order to control for potentially deleterious effects, the host cell inhibits retrotransposon expression through various mechanisms, including extensive DNA methylation. Retrotransposons can escape this inhibition during germ cell development due to the global erasure of genomic DNA methylation. Therefore, retrotransposition events are more frequent in germ cells and in early stages of embryo development compared with somatic cells^29^ increasing the risk of de novo mutations. The insertion described here could have occurred during germline development in the maternal grandparents or early during maternal embryonic development.

This is the third report of patients with AIS and mutations in the *AR* 5′UTR^3,24^. We therefore recommend to include the *AR* 5′UTR in the genetic analysis of the *AR* in patients with a clear clinical diagnosis of AIS. A better understanding of the regulation of *AR* expression through its 5′UTR is necessary to uncover unknown etiologies of AIS and to better understand the transcriptional and translational regulation of the *AR*.

## Methods

The study was performed with approval of the Ethical Committee of the Christian-Albrechts-University, Kiel, Germany (AZ: D552/16). We obtained written consent from the parents on behalf of the children enrolled in this study. We included scrotal biopsies of patients under the age of 18 years who underwent orchidopexy due to maldescended testes with normal external genitalia, i.e., no hypospadias as controls.

### Cell culture

GSFs were grown in phenol red free Dulbecco's modified Eagle's medium (Life Technologies) supplemented with 10% fetal bovine serum (FBS; Maxspec, Gibco), 100 units/ml penicillin/streptomycin, 2 mM l-glutamine (Biochrome) and 20 mM HEPES buffer (Life Technologies) at 37 °C with 5% CO_2_. Hormone induction experiments were done as previously described^22^. Briefly, 1.2 × 10^5^ cells were plated in 6 cm dishes in complete tissue culture medium. After 24 h cells were washed three times with the above-described medium without FBS, then a complete tissue culture medium containing 10% charcoal-treated FBS was added to the cells. To one dish DHT (Sigma-Aldrich, Germany) dissolved in 100% ethanol was added to a final concentration of 10 nM. An equal volume of ethanol was added to the second dish. Cells were left for 72 h under these conditions at 37 °C with 5% CO_2_ after which they were lysed in RNA-extraction buffer (RLT; Qiagen). Protein lysates were taken 48 h after hormone treatment.

List of GSFs used:

**Table:** 

ID	Patient/control	Age at biopsy	Tissue of origin
GSF-37	Male control 1	3 years	Scrotal skin
GSF-41	Male control 2	1 year	Scrotal skin
GSF-172	Patient	1 year 8 months	Labioscrotal skin

### Nucleic acid extraction

Standard DNA extraction was performed with DNeasy Blood and Tissue kit (Qiagen) from genital skin fibroblasts, according to the manufacturer's instructions. High molecular weight (HMW) DNA was extracted from genital skin fibroblasts with Nanobind CBB kit (PacBio), according to the manufacturer's instructions, and it was further concentrated using a SpeedVac vacuum concentrator prior to its use for long-read sequencing. RNA extraction was performed using the RNeasy Mini Kit (Qiagen) following the manufacturer's instructions.

### Whole exome sequencing (WES)

After routine diagnostic referral, the exome of the patient was enriched using the SureSelectXT Clinical Research Exome V2 (Agilent, elid S30409818, genome build GRCh37) and sequenced on a Illumina Novaseq 6000 sequencer. The sequencing data was processed with an in-house developed pipeline^30^. Since the *AR* gene was highly suspect, analysis was only performed on this gene. Analysis was performed using Alissa Interpret software (Agilent). This analysis showed a putative deletion in the promoter region that was not previously seen in controls. Manual inspection of the .bam file showed a mapping pattern consistent with a retrotransposon insertion, explaining the deletion call. Blast analysis of linked reads suggested that this insertion could be a L1 insertion. This insertion was confirmed with PCR.

### Whole genome sequencing (WGS)

Library preparation was performed on blood-derived DNA samples from the patient and parents (trio), with an Illumina DNA Prep Tagmentation kit, according to the manufacturer's protocol. For each library, concentration was measured by Qubit dsDNA-HS (High Sensitivity) assay (Invitrogen) and fragment size distribution was checked by Bioanalyzer 2100 (Agilent High Sensitivity DNA kit) to estimate library molarity. The library pool was created using an equimolar amount of each sample, and the library pool was quantified by qPCR using the NEBNext Library Quant Kit for Illumina. The Library pool was finally sequenced PE 2*150 cycles on a S4 flow cell, on a NovaSeq 6000 device (Illumina). Sequencing data was demultiplexed and converted into fastq format using bcl2fastq2 v2.20 and aligned against the 1000 genomes Reference Genome Sequence (hs37d5) for SNP and Indel calling using the GATK4 tool kit. Delly^31^, Manta^32^ and LUMPY^33^ tool kits were used to identify structural variants. Variants were filtered and prioritized using VarFish^34^.

### Cloning

In order to investigate effects of the L1 insertion on *AR* expression, different constructs were created using Gibson Assembly strategy. pcDNA3.1 plasmid containing the wild type *AR* 5′ UTR as well as the *AR* coding DNA (cDNA) sequence was available from our previous study^3^. This plasmid was first linearized by PCR, and then either the L1 sequence (amplified from patient-derived DNA from genital skin fibroblasts) or a size-matched control (amplified from a healthy control DNA) was inserted in the *AR* 5′ UTR. Primer sequences are as follows:

**Table:** 

	Forward primer sequence	Reverse primer sequence
L1 cloning		
Primers to linearize the plasmid	CCCCCGTCGGCCCAGCGCTGCC	GGGCTGGCGTGGTGCGTCCCTTCG
Primers to amplify the L1 sequence	CGAAGGGACGCACCACGCC	CTGGCAGCGCTGGGCCGAC
Size-matched control cloning		
Primers to linearize the plasmid	GTAACTTCTCTGTTGAaaaagctgctaaagactcggag	CAACGAGAGTGAGCTTGaaaaacttcaccgaagaggaaag
Primers to amplify the control sequence (*AR* intron 1)	CAAGCTCACTCTCGTTGGC	TCAACAGAGAAGTTACCATCCC

To confirm that no undesired mutations were created in the cloning process, Nanopore whole-plasmid sequencing was performed by the company Eurofins. Mutation-free clones were amplified, plasmid DNA was extracted with Plasmid Plus Midi kit (Qiagen), and used for downstream experiments.

### Transfection and RNA and protein extraction

Human Embryonic Kidney (HEK) 293 cells were grown in Dulbecco’s Modified Eagle’s medium (Life Technologies) supplemented with 10% fetal bovine serum (FBS superior; Sigma), 100 units/ml penicillin/streptomycin, and maintained in a tissue culture incubator at 37 °C with 5% CO_2_ until they reached 90% confluency. The cells were seeded at 2.5 × 10^5^ cells per well in a 6 well cell culture plate. After a 24 h incubation in growth medium, cells were transfected with 1 μg of plasmid DNA using Lipofectamine 3000 according to the manufacturer’s instructions (Invitrogen). Following 24 h of transfection, cells were lysed with RLT buffer (Qiagen) and RNA was extracted using RNeasy Mini Kit (Qiagen). p-values were calculated using a two-sided t-test.

For protein extraction cells were seeded at 2.5 × 10^5^ cells per well in a 12 well cell culture plate, and transfection was done using Lipofectamine 3000 according to manufacturer’s instructions (Invitrogen). After 6 h of transfection, the medium was removed and the cells were washed two times in phenol red-free Dulbecco’s modified Eagle medium and further grown in the same medium supplemented with 0.1% charcoal treated FBS (serum starved conditions). Cells were treated with non-aromatizable DHT dissolved in 100% ethanol at a final concentration of 10 nM or an equal volume of ethanol (EtOH) and incubated under the described conditions at 37 °C with 5% CO_2_ for 48 h. Whole cell protein lysates were obtained by resuspending the cells in ice-cold RIPA buffer, supplemented with a cocktail of protease inhibitors (CompleteTM, Roche).

### RNA detection

500 ng of total RNA was reverse transcribed using the QuantiTect Reverse Transcription Kit (Qiagen). Quantitative PCR was performed with the QuantiTect SYBR Green master mix (Qiagen) using primers against *APOD*, *AR* and *SDHA*. All primers were purchased from Qiagen and used following the manufacturer's instructions. For HEK293T transfections, 500 ng RNA was treated twice with TURBO DNase (Life Technologies) to eliminate residual plasmid DNA and reverse transcribed using the QuantiTect Reverse Transcription Kit (Qiagen). The reverse transcription reaction was also performed in the absence of Reverse Transcriptase and gave no amplification signal indicating the complete digestion of plasmid DNA. Quantitative PCR was performed with the QuantiTect SYBR Green master mix (Qiagen) using primers against *AR* and the neomycin resistance gene (neo)^3^. p-values were calculated using a two-sided *t*-test.

### Protein detection

Protein extracts were separated by SDS-PAGE on a NuPAGE 4% to 12% Bis–Tris gel (Invitrogen) and transferred to a nitrocellulose membrane (Amersham Protran 0.45 μm NC). The membrane was blocked for 1 to 2 h at room temperature with Tris-buffered saline containing 0.1% Tween-20 and 5% nonfat dry milk. For AR, Actin and Tubulin detection, anti-AR F39.4.1 antibody (BioGenex, 1:200 dilution), anti-Actin (ab 8227 abcam, 1:8000 dilution) and anti-alpha Tubulin antibody (T5168, Sigma, 1:10,000 dilution) was used respectively overnight at 4 °C. Secondary antibodies (Cell Signaling Technology and Immunoreagents) were incubated for 75 min at room temperature (1:5000 dilution). The antibodies were resuspended in Tris-buffered saline containing 0.1% Tween and 5% nonfat dry milk. Signals were detected with the Biorad Chemidoc imaging system using the Immobilon Forte Western HRP Substrate (Merck Millipore).

### Cas9-mediated target enrichment

Cas9-targeted sequencing from Oxford Nanopore Technologies (ONT) was performed to enrich the target region and to obtain the full-length L1 sequence as well as epigenetic information. CRISPR RNAs (crRNAs) were designed with CHOPCHOP (https://chopchop.cbu.uib.no (accessed on 11 October 2023)). Two crRNAs were used upstream of the *AR* L1 insertion, and two crRNAs were used downstream. The enriched DNA was prepared with the Nanopore Ligation Sequencing Kit (SQK-LSK110 and SQK-LSK114), loaded on R10.4.1 flow cells and sequenced with GridION. Basecalling was performed with guppy (version 6.4.2), and quality of reads was analyzed with the software Nanostat (version 1.5.0). Alignment to the human genome (hg38) was performed with Minimap2 (version 2.22). To counteract potential off-target CRISPR/Cas9 enrichment effects, BAM files were filtered for reads with an alignment length > 3 kb in the patient or > 1.5 kb in control samples. Methylation was called with the dna_r10.4.1_e8.2_260bps_modbases_5mc_cg_sup.cfg of guppy (version 6.4.2). Only CpG sites covered by > 10 reads were included in the analysis.

Sequence similarity alignment of the sequenced L1 element was done using the basic local alignment search tool balstx (NCBI).

### Supplementary Information

Supplementary Information.

## Data Availability

Raw sequencing data is currently being submitted to GEO database but can be made available by the authors on request for the purpose of review.
