# Supplementary material for: LINE1-mediated epigenetic repression of androgen receptor transcription causes androgen insensitivity syndrome
Source: Sci Rep. 2024 Jul 15;14:16302. doi: 10.1038/s41598-024-65439-w (PMC11251026; doi:10.1038/s41598-024-65439-w)

LINE1-mediated epigenetic repression of androgen receptor transcription causes androgen insensitivity syndrome

Supplementary Information

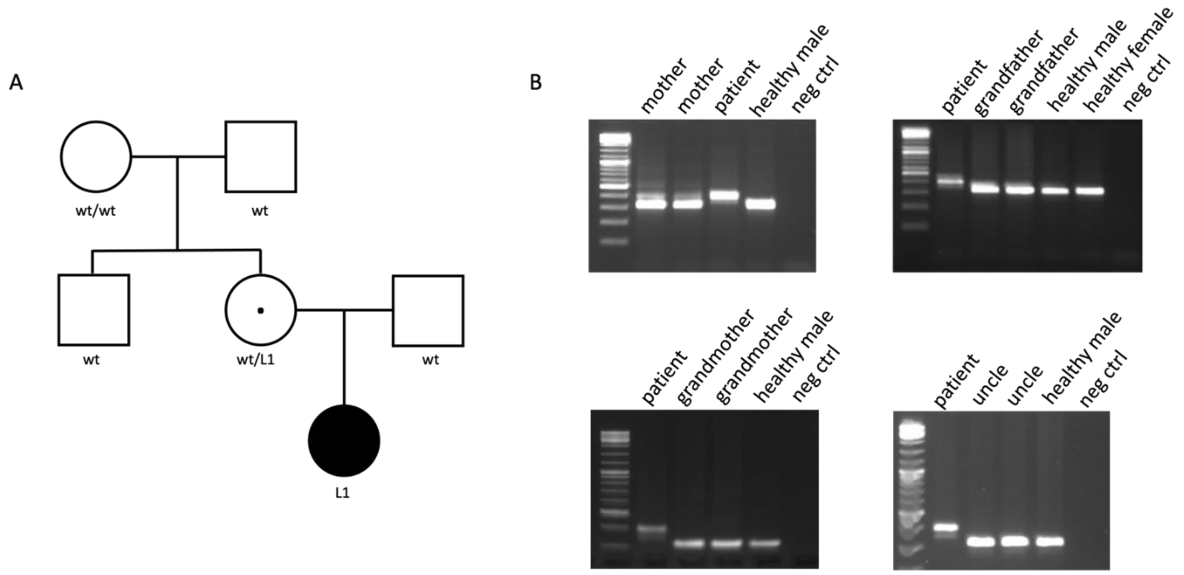

Supplementary Figure 1. Segregation of the L1 insertion in the family. (A) pedigree showing the patient (46,XY but raised as a girl), the unaffected mother (carrier) and her family. (B) PCR on blood DNA from the patient (396bp), different maternal family members and healthy controls (314bp).

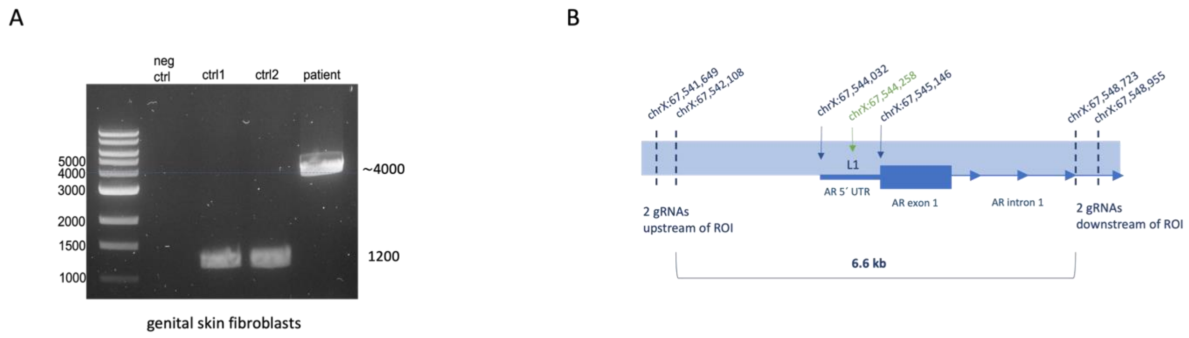

Supplementary Figure 2. Genetic analysis in patient-derived cells. (A) long-range PCR showing an estimated length of the L1 insertion in patient-derived cells, as compared to wild type bands in two healthy controls. (B) experimental design of Cas9-mediated target enrichment, showing target locations of gRNAs upstream and downstream of the region of interest (ROI), along with genomic coordinates in the hg38 assembly.

Supplementary File 1

TCGGTGAAGTTTTTTTTTTTTTTTTTTTTTTTTTTTTTTTTTTTTTTTTTTTTTTTATTATACTCTAAGTTTTAGGGTA
CATGTGCACATTGTGCAGGTTAGTTACATATGTATACATGTGCCATGCTGGTGCACTGCACCCACTAATGTGTC
ATCTAGCATTAGGTATATCTCCCAATGCTATCCCTCCCCCCTCCCCCGACCCCACCACAGTCCCCAGAGTGTGAT
ATTCCCCTTCCTGTGTCCATGTGATCTCATTGTTCAATTACCACCTATGAGTGAGAATATGCGGTGTTTGGTTTT
TTGTTCTTGCGATAGGTTACTGAGAATGATGGTTTCCAATTTCATCCATGTCCCTACAAAGGATATGAACTCAT
CATTTTTTATGGCTGCATAGTATTCCATGGTGTATATGTGCCACATTTTCTTAATCCAGTCTATCATTGTTGGGC
ATTTGGGTTGGTTCCAAGTCTTTGCTATTGTGAATAGTGCCGCAATAAACATACGTGTGCATGTGTCTTTATAG
CAGCATGATTTATACTCATTTGGGTATATACCCAGTAATGGGATGGCTGGGTCAAATGGTATTTCTAGTTCTAG
ATCCCTGAGGAATCGCCACACTGACTTCCACAATGGTTGAACTAGTTTACAGTCCCACCAACAGTGTAAAAGT
GTTCCTATTTCTCCGCATCCTCTCCAGCACCTGTTGTTTCCTGACTTTTTAATGATTGCCATTCTAACTGGTGTGA
GATGATATCTCATAGTGGTTTTGATTTGCATTTCTCTGATGGCCAGTGATGATGAGCATTTCTTCATGTGTTTTT
TGGCTGCATAAATGTCTTCTTTTGAGAAGTGTCTGTTCATGTCCTTCGCCCACTTTTTGATGGGGTTGTTTGTTT
TTTTCTTGTAAATTTGTTTGAGTTCATTGTAGATTCTGGATATTAGCCCCTTTGTCAGATGAGTAGGTTGCAAAA
ATTTTCTCCCATGTTGTAGGTTGCCTGTTCACTCTGATGGTAGTTTCTTTTGCTGTGCAGAAGCTCTTTAGTTTA
ATTAGATCCCATTTGTCAATTTTGTCTTTTGTTGCCATTGCTTTTGGTGTTTTGGACATGAAGTCCTTGCCCACGC
CTATGTCCTGAATGGTAATGCCTAGGTTTTCTTCTAGGGTTTTTATGGTTTTAGGTTTAACGTTTAAATCTTTAA
TCCATCTTGAATTGATTTTTGTATAAGGTGTAAGGAAGGGATCCAGTTTCAGCTTTCTACATATGGCTAGCCAG
TTTTCCCAGCACCATTTATTAAATAGGGAATCCTTTCCCCATTGCTTGTTTTTCTCAGGTTTGTCAAAGATCAGA
TAGTTGTAGATATGCGGCATTATTTCTGAGGGCTCTGTTCTGTTCCATTGATCTATATCTCTGTTTTGGTACCAG
TACCATGCTGTTTTGGTTACTGTAGCCTTGTAGTATAGTTTGAAGTCAGGTAGTGTGATGCCTCCAGCTTTGTT
CTTTTGGCTTAGGATTGACTTGGCAATGCGGGCTCTTTTTTGGTTCCATATGAACTTTAAAGTAGTTTTTTCCAA
TTCTGTGAAGAAAGTCATTGGTAGCTTGATGGGGATGGCATTGAATCTGTAAATTACCTTGGGCAGTATGGCC
ATTTTCACGATATTGATTCTTCCTACCCATGAGCATGGAATGTTCTTCCATTTGTTTGTCTCCTCTTTTATTTCCTT
GAGCAGTGGTTTGTAGTTCTCCTTGAAGAGGTCCTTCACATCCCTTGTAAGTTGGATTCCTAGGTATTTTATTCT
CTTTGAAGCAATTGTGAATGGGAGTTCACCCATGATTTGGCTCTCTGTTTGTCTGTTGTTGGTGTATAAGAATG
CTTGTGATTTTTGTACATTGATTTTGTATCCTGAGACTTTGCTGAAGTTGCTTATCAGCTTAAGGAGATTTTGGG
CTGAGACGATGGGGTTTTCTAGATAAACAATCATGTCGTCTGCAAACAGGGACAATTTGACTTCCTCTTTTCCT
AATTGAATACCCTTTATTTCCTTCTCCTGCCTGATTGCCCTGGCCAGAACTTCCAACACTATGTTGAATAGGAGT
GGTGAGAGAGGGCATCCCTGTCTTGTGCCGGTTTTCAAAGGGAATGCTTCCAGTTTTTGCCCATTCAGTATGA
TATTGGCTGTGGGTTTGTCATAGATAGCTCTTATTATTTTGAAATACATCCCATCAATACCTAATTTATTGAGAG
TTTTTAGCATGAAGGGTTGTTGAATTTTGTCAAAGGCTTTTTCTGCATCTATTGAGATAATCATGTGGTTTTTGT
CTTTGGCTCTGTTTATATGCTGGATTACATTTATTGATTTGCGTGTATTGAACCAGCCTTGCATCCCAGGGATGA
AGCCCACTTGATCATGGTGGATAAGCTTTTTGATGTGCTGCTGGATTCGGTTTGCCAGTATTTTATTGAGGATT
TTTGCATCAATGTTCATCAAGGATATTGGTCTAAAATTCTCTTTTTTTGGTTGTGTCTCTGCCCGGCTTTGGTATC
AGTATGATGCTGGCCTCATAAAATGAGTTAGGGAGGATTCCCTCTTTTTCTATTGATTGGAATAGTTTCAGAAG
GAATGGTACCAGTTCCTCCTTGTACCTCTGGTAGAATTCGGCTGTGAATCCATCTGGTCCTGGACTCTTTTTGG
TTGGTAAACTATTGATTATTGCCACAATTTCAGAGCCTGTTATTGGTCGATTCAGAGATTCAACTTCTTCCTGG

Supplementary File 1. Sequence of the L1 insertion obtained from nanopore sequencing. Bases marked in gray represent the genomic DNA sequence proximal to the L1 insertion.

Uncropped images

Western blot image from Figure 1:

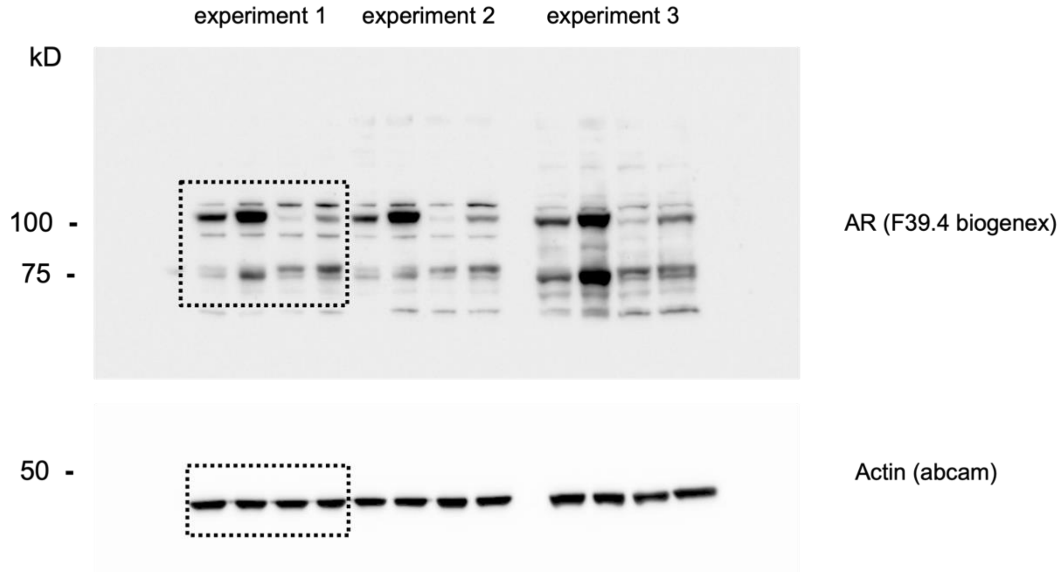

Membrane was cut after blotting between 50 kD and 75 kD. The upper part was incubated with the anti-AR antibody F39.4 from Biogenex, the lower part was incubated with the anti-actin antibody from Abcam. Shown are three biological replicates. Dotted lines indicate the selection taken for Figure 1.

Western blot image from Figure 3:

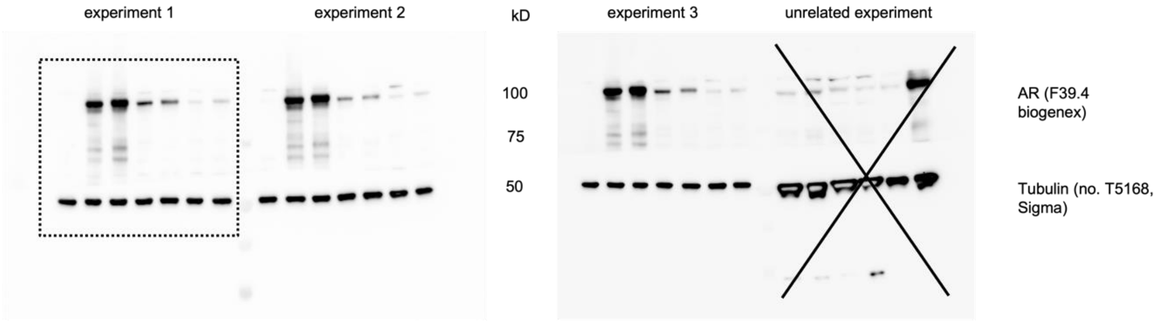

Membrane was cut after blotting between 50 kD and 75 kD. The upper part was incubated with the anti-AR antibody F39.4 from Biogenex, the lower part was incubated with the anti-tubulin antibody from Sigma. Shown are three biological replicates. Dotted lines indicate the selection taken for Figure 3.

Agarose gel images from Supplementary Figure 1 were generated in a diagnostic laboratory according to ISO15189 guidelines.

Agarose gel from Supplementary Figure 2:

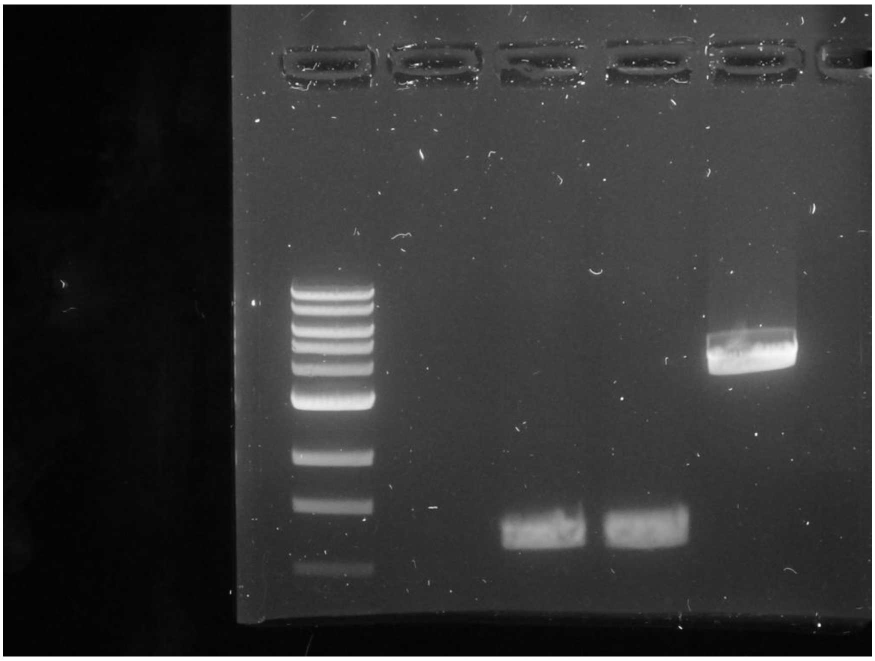

Supplement: Supplementary file 1 — Supplementary Information. [file 41598_2024_65439_MOESM1_ESM.pdf]
